# HLA Mismatching Strategies for Solid Organ Transplantation – A Balancing Act

**DOI:** 10.3389/fimmu.2016.00575

**Published:** 2016-12-07

**Authors:** Andrea A. Zachary, Mary S. Leffell

**Affiliations:** ^1^Department of Medicine, Johns Hopkins University School of Medicine, Baltimore, MD, USA

**Keywords:** HLA matches, HLA mismatches, immunogenicity, match probability, sensitization, repeated mismatches, donor-specific antibody

## Abstract

HLA matching provides numerous benefits in organ transplantation including better graft function, fewer rejection episodes, longer graft survival, and the possibility of reduced immunosuppression. Mismatches are attended by more frequent rejection episodes that require increased immunosuppression that, in turn, can increase the risk of infection and malignancy. HLA mismatches also incur the risk of sensitization, which can reduce the opportunity and increase waiting time for a subsequent transplant. However, other factors such as donor age, donor type, and immunosuppression protocol, can affect the benefit derived from matching. Furthermore, finding a well-matched donor may not be possible for all patients and usually prolongs waiting time. Strategies to optimize transplantation for patients without a well-matched donor should take into account the immunologic barrier represented by different mismatches: what are the least immunogenic mismatches considering the patient’s HLA phenotype; should repeated mismatches be avoided; is the patient sensitized to HLA and, if so, what are the strengths of the patient’s antibodies? This information can then be used to define the HLA type of an immunologically optimal donor and the probability of such a donor occurring. A probability that is considered to be too low may require expanding the donor population through paired donation or modifying what is acceptable, which may require employing treatment to overcome immunologic barriers such as increased immunosuppression or desensitization. Thus, transplantation must strike a balance between the risk associated with waiting for the optimal donor and the risk associated with a less than optimal donor.

## Introduction

There is overwhelming evidence of the benefits of HLA matching in organ transplantation including better graft function, longer graft and patient survival, and reduced risk of sensitization. However, when a well-matched related donor is not available, the wait for a well-matched unrelated donor can be prolonged, which can reduce quality of life, impede physical and cognitive development in the young, and increase the risk of death. Furthermore, in countries where there is substantial ethnic diversity, allocation of deceased donor organs by HLA match can result in a disparity, among ethnic groups, in access to transplantation. The effects of HLA matching are confounded by many factors that can affect outcome such as sensitization, immunosuppression, recipient ethnicity and age, and donor type and quality. Thus, transplantation is a balancing act between capturing the benefits of a well-matched transplant and diminishing the problems associated with achieving that transplant. Strategies must consider both the benefits and disadvantages of matching, the detrimental effects of mismatching, and what can be done to minimize negative effects of both matching and mismatching.

Here, we will review the impact of HLA matching/mismatching on graft outcomes, other factors that impact the effect of HLA, other consequences of mismatches, and the factors that should be evaluated – HLA antigens, epitopes, and amino acids. We will examine the effect of HLA mismatches on the current transplant and on future transplants as well as HLA matching strategies for the non-sensitized and sensitized patients.

## Effect of HLA Matching/Mismatching on Outcomes

Assessment of the effects of mismatching has been confounded by variability over time of the ability to determine HLA phenotype accurately; by considering only matched but not mismatched antigens; by evaluating the effect of only some HLA loci; and by the diminished sensitivity and specificity of cell-based tests for HLA antibody. Although numerous early studies reported that increased numbers of matched antigens or decreased numbers of mismatched antigens led to improved graft and patient survival, improved graft function, and fewer rejection episodes, later reports suggested that ongoing improvements in immunosuppression therapies either diminished or eliminated any benefit of matching. However, large studies and more recent reports have reaffirmed the benefits to be derived from matching. Data from the Collaborative Transplant Study showed that with or without cyclosporine use, the renal transplant success rate was 20% higher when there was no mismatch of HLA-B and -DR than when there was a mismatch (1). Similarly, data from the United Network for Organ Sharing showed that long-term graft survival of deceased donor renal transplants with no HLA-A, -B, and -DR mismatch was nearly 20% better than for fully mismatched grafts with a stepwise reduction in survival with each increased degree of mismatch (2). Similar results were observed in a study of more than 150,000 renal transplants in which 10-year graft survival of first deceased donor kidney transplants was 17% higher among the zero HLA-A, -B, and -DR-mismatched patients than among those fully mismatched with an even greater benefit derived in sensitized patients (PRA >50%) (3). When graft survival was examined for deceased donor renal transplants occurring in different eras, it was seen that 5-year graft survival was 11% higher among transplants occurring between 1995 and 2004 compared to those occurring in the 10 years prior (73 vs. 62%) and that the strength of the association with HLA mismatch decreased in the second decade, but was still present. Furthermore, an association between extent of mismatch and treatment for rejection was present in both decades (4). In contrast, Su et al. found a diminishing benefit of HLA matching in deceased donor renal transplants over the period 1995–1998 (5). A single center study showed a dramatic benefit of HLA matching among highly sensitized patients receiving deceased donor kidney transplants (6). One hundred and forty-two patients with CPRA >80%, negative flow cytometric crossmatches (FCXM) with donor T and B lymphocytes, and no detectable donor-specific antibody tested by ELISA, were grouped according to mismatch. For patients with 0–2, 3–4, or 5–6 HLA-A, -B, and -DR mismatches, the incidence of rejection was 4.4, 11.4, and 31.3% and 5-year graft survival was 100, 81, and 74%, respectively. This study found a strong effect of HLA-A, but not -B or -DR mismatch on graft loss. Others have found that mismatches for class I and class II had independent effects on patient survival where 0-DR/2-4AB and 0-1AB/1-2DR mismatches had 10-year patient survival of 86 and 89%, respectively, compared to only 74% for 1-4 AB/1-2DR mismatches. The best survival of 92% was with mismatches limited to 1 A or B antigen. Freedom of graft failure due to immunologic causes was 96.5% for mismatches limited to 1 A or B antigen and no DR mismatch and was 89–91% for all other mismatch groups (7).

**Figure 1:**
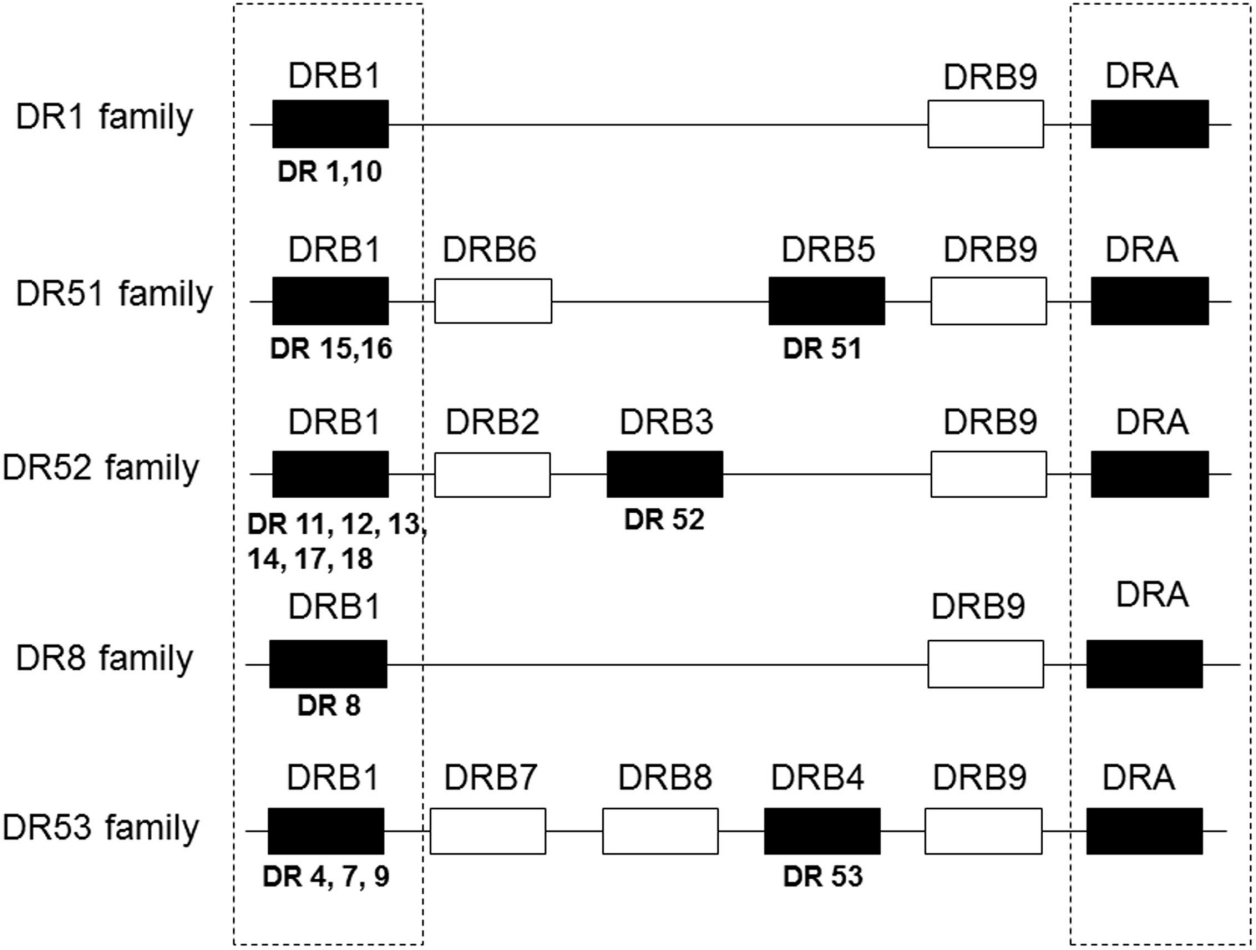
**Gene organization of HLA-DR haplotypes**. DR haplotypes have varying numbers of genes, some of which encode a polypeptide chain (filled boxes) and others are pseudogenes that have no detectable product (open boxes). DR molecules are comprised of two polypeptide chains, an α chain and a β chain. All DR haplotypes have a DRA gene that encodes the relatively invariant α chain and a DRB1 gene that encodes the β chain of the DR1-DR18 antigens. Some haplotypes carry an additional gene, DRB3, 4, or 5, that encodes the β chain of the DR52, 53, or 51 molecules, respectively. DR haplotypes can be grouped into families defined by the number of DRB genes present as shown in the diagram.

Although there had been reports of the role of HLA-A and/or -B mismatch, it eventually became apparent that of the HLA antigens tested routinely, HLA-DR matching contributed the most to graft survival and function. This is of particular importance since there are fewer antigens encoded by the DRB1 locus than by either the A or B loci making it easier to find zero DR mismatches compared to zero A or B mismatches, particularly when dealing with a very HLA heterogeneous population as in the United States. Connolly et al. (8) showed that among 516 primary deceased donor kidney recipients, zero DR-mismatched transplants had significantly better survival than those with even a single DR mismatch at both 1 year (92.8 vs. 84.5%) and 5 years (88.3 vs.73.9%) (*P* < 0.0001). The effect was independent of HLA-A or -B match but diminished if cold ischemia time was more than 26 h. Al-Otaibi et al. (9) found that pediatric renal patients who received fully DR-matched grafts had significantly better graft survival than did those receiving grafts with one or two DR mismatches. However, in this study, there were more living donors in the well-matched group, which most likely contributed to the outcomes. It is not clear why matching for DR would be more important than matching for HLA-A or -B. Perhaps, DR antigens are more immunogenic than are A or B antigens. Perhaps, there is a gene dose effect. There is strong linkage disequilibrium within the HLA complex such that mismatching for DR may also increase the likelihood of mismatching for DQ antigens that have not been included in many evaluations of associations between match and outcome. Additionally, mismatching for DRB1 antigens may also include a mismatch for the antigens encoded by the linked DRB3, 4, and 5 loci that encode DR52, 53, and 51, respectively. Figure 1 shows that most DR haplotypes are fixed such that DR15 or 16 also have the DRB5 gene that encodes DR51. Haplotypes with DR11, 12, 13, 14, 17, or 18 bear the DRB3 gene that encodes DR52 and haplotypes with DR4, 7, or 9 have the DRB4 gene that encodes DR53. (Note that some very rare DR1 haplotypes also have the DRB5 gene. Also, an exception occurs on haplotypes bearing DR7 and DQ9. These haplotypes have a null allele at the DRB4 locus and do not express DR53.) So that a patient with a DR1, DR4 phenotype, who is mismatched with a DR11, 12, 13, 14, 17, or 18, is also mismatched for DR52. To complicate matters further, DR52 antigens share an epitope with DR11, 12, 13, 14, 17, and 18 (10) and with DR8 (11) so that in addition to two mismatched antigens, there is a double dose of the shared epitope. Indirect evidence to support the possibility of a gene dose effect was reported by Kim et al. (12) who assessed graft survival in patients mismatched for one or more antigens present in either the heterozygous state (one copy of the antigen) or homozygous state (two copies of the mismatched antigen) with the latter being scored as a single mismatch in most studies. They found that zygosity affected both mean and 10-year survival with worse outcomes occurring with two doses of the mismatched gene (Table 1). In the past decade, there have been numerous, additional reports correlating improved outcomes with reduced mismatches of HLA-A, -B, and/or -DR antigens (13–19), and there has been little or no evidence to the contrary.

**Table 1:** **Effect of gene dose of mismatched antigen**.

Mismatched antigen	Mean graft survival (years)	
	Heterozygous	Homozygous
1 HLA-B	20.1	6.7
1 HLA-DR	16.9	14.7
1 each HLA-A, -B, -DR	15.0	13.0
	**10-year graft survival (%)**	
1 HLA-B	85	0
1 HLA-DR	87	67
1 each HLA-A, -B, and -DR	84	70

*Homozygosity with two copies of a mismatched antigen, compared to heterozygosity with a single copy, was associated with reduced mean graft survival and 10-year graft survival. Adapted from Kim et al. (12)*.

Nearly, all studies have examined outcomes vis-a-vis matching or mismatching of HLA-A, -B, and -DRB1-encoded antigens. However, at the time of this writing, there are limited data on matching at other HLA loci (HLA-C, -DRB3, -DRB4, -DRB5, -DQA, -DQB, -DPA, and -DPB). Frohn et al. investigated the impact of HLA-C mismatches on rejection in 104 renal transplants (20). They controlled for HLA-B mismatch to eliminate linkage disequilibrium as a confounding factor. They found that patients with one or two mismatches for an HLA-C antigen had a significantly higher incidence of rejection compared to those with no HLA-C mismatch (54 and 100 vs. 0%) but only when there was also one HLA-B mismatch. Patients with one HLA-B and two HLA-C mismatches also had decreased graft survival that approached statistical significance (*P* = 0.055). In an early study of data from the Southeastern Organ Procurement Foundation on 12,050 first deceased donor transplants, no effect of matching for HLA-DQ was found when other factors affecting outcome were taken into account (21). In contrast, in a recent study, Lim et al. found DQ mismatching to incur a significantly increased risk of rejection that was further increased in the presence of DR mismatches (22). Rosenberg et al. (23) found that DP mismatches in patients matched for DR and DQ did not impact graft survival or function. Similarly, data from the Collaborative Transplant Study found no deleterious effect of DP mismatches in first deceased donor transplants but did find significantly reduced graft survival in regraft patients (24). The widespread adoption of DNA-based typing methods has facilitated typing for HLA-DQ and -DP. However, unlike other HLA molecules that have one polypeptide chain that is invariant or has limited variability, both polypeptide chains, the α chain encoded by DQA and DPA and the β chain encoded by DQB and DPB, are polymorphic. Both chains may have immunogenic epitopes and there are epitopes comprised of particular combinations of α and β chains (25, 26). Consequentially, studies that consider matching only for DQB and DPB may incorrectly identify a mismatch as a match between a donor and recipient.

The benefits of matching have been seen in all or nearly all types of transplants defined by organ type including heart (27–32), lung (33–36), liver (37–40), and pancreas (41, 42). It should be noted, however, that there are numerous reports of a lack of an effect of HLA matching on outcomes in liver transplantation. The production of large amounts of soluble HLA class I molecules and the dual vasculature of the liver may reduce the susceptibility of this organ to immune attack. Regarding pancreas transplantation, most studies of matching have been of simultaneous kidney–pancreas transplants. The two citations here are of pancreas only transplants.

### Other Effects of HLA Mismatches

In separate reports from the Collaborative Transplant Study, it was shown that HLA mismatches were associated with death with functioning graft and with posttransplant lymphoproliferative disease. Increasing numbers of HLA mismatch in renal transplantation were associated with an increased need for antirejection therapy that might account for an increased incidence of death with functioning graft due to infection and cardiovascular disease (43). It was also shown that among 9,209 pediatric kidney transplants, HLA-A, -B, and -DR mismatches were a risk factor for 5-year graft survival, but two DR mismatches appeared to incur an increased risk for non-Hodgkins lymphoma (44). There is a differential cost associated with different degrees of HLA match. Schnitzler et al. looked at Medicare payment information in the United States Renal Data System (USRDS) according to the degree of HLA mismatch. At three years, Medicare payments for zero, 1–3, 4–5, and 6 antigen mismatches were $60,400, $64,000, $71,000, and $81,000, respectively (45). Increased sensitization associated with HLA mismatches and HLA matching strategies are topics requiring more extensive discussion and will be discussed below.

## Other Factors Related to Matching and Outcomes

### Regrafts

Coupel et al. (46) reported on 233 second renal transplants for which repeated mismatches were permitted when no antibody to the mismatch was present. They found that DR mismatch was a major predictor of graft loss with DR mismatched patients having 5- and 10-year graft survival rates of 73 and 54%, respectively, compared to 82 and 69% for zero DR mismatches. Others have investigated if there was a risk associated with repeated mismatches in regrafts. Cabacungan (47) found no effect of a repeated class I (HLA-A, -B) mismatch, but saw a 5–8% decrease in 1-year graft survival when there was a repeated DR mismatch. This is in contrast to a report of transplants occurring in Southeastern Organ Procurement Foundation member centers, which found a lack of risk associated with repeated mismatch occurring in 158 of 753 regrafts (48). Doxiadis et al. (49) found that repeated DR mismatches, but not repeated A or B mismatches, significantly reduced 1-, 3-, and 5-year graft survival and that the effect was magnified when the survival of the first graft was less than 6 months. Tinckam et al. (50) looked at the effect of a repeated mismatch present in 3,868 of 13,789 regrafts listed in the USRDS. They found that repeated class I or class II mismatches were a risk for graft loss in patients who underwent transplant nephrectomy of the first transplant prior to receiving the second transplant and that the effect was stronger with class II mismatches. Repeated class II mismatches were also a risk factor in sensitized patients. They postulated that sensitization may be a marker for a more aggressive responder type or an indication of undetected low-level antibody to the repeated mismatch. Risk of graft loss was limited to those two subgroups of patients, sensitized patients, and patients who had undergone nephrectomy of the previous transplant. This report is extremely important because avoiding repeated mismatches unnecessarily reduces a patient’s chance for transplantation and increases waiting time while increasing immunosuppression in the face of a repeated mismatch may be unnecessary and incur an increase in the attendant side effects. Additional studies could determine further the level of risk associated with repeated mismatches, particularly among patients who are neither sensitized nor have had a nephrectomy of a previous transplant.

### Recipient Race

Historically, Black recipients were considered to have poorer survival of renal grafts compared to White recipients. However, there are limited data on potentially combined effects of HLA matching and recipient race. Butkus et al. (51) showed that the effect of HLA mismatching in deceased donor transplantation was comparable in Blacks and Whites but that Blacks had, on average, more antigen mismatches. They found that the poorer graft survival among Black patients was attributable to socioeconomic factors, such as the type of insurance coverage and non-compliance. Smith and Butterly (52) saw a disparity between Black and White recipients of living donor transplants at all levels of match but that the disparity was diminishing over time from 1985 to 2000.

### Donor Factors

Donor factors, such as age and type, may exacerbate or reduce the effect of HLA mismatches. Using USRDS data for pediatric renal transplants occurring during 1994–2004, Foster et al. (53) examined the effect of HLA mismatch with consideration of donor age and further categorized transplants by donor type (deceased or living). Donors were grouped by age in 5-year increments up to the age of 50 with donors older than 50 comprising the last group. They found that, among deceased donor transplants, there was a significant difference in graft survival between the best- and worst-matched transplants with each increasing number of mismatches increasing the risk of graft failure by 4% with donors 35 years old or older. Among the deceased donor transplants, young donor age offset the impact of poorer matches while better matches ameliorated the negative impact of older donor age. Among living donor transplants, they found that HLA mismatch, but not donor age, was relevant to graft survival and that 5-year graft survival was better among poorly matched living donor transplants than among well-matched transplants with deceased donors of any age. Similarly, Terasaki et al. (54) reported that 10-year graft survival was better with five to six mismatched antigens when the donor was young than with zero mismatches and donor age greater than 55 years. A study of risk factors among 1,632 living donor renal transplants found that risk factors for patient survival were donor >65 years old and five to six antigen mismatch, while risk factors for graft survival were donor >65 years old and a mismatch of three antigens or more (55).

### Immunosuppression

It was believed that the development and use of cyclosporine and lymphocyte depleting agents would diminish or negate the effect of HLA on outcomes, but the impact was limited. However, there is currently a wide array of therapeutic agents used for induction, maintenance immunosuppression, and/or treatment of rejection, and these may have a more substantial impact on the effect of HLA mismatches. In 2001, Meier-Kriesche et al. (56) compared the effects of mycophenolate mofetil (MMF) and azathioprine on matching in 8,459 and 11,216 first renal transplants. They found that there was less graft loss with MMF than with azathioprine, but that there was still a significantly lower rate of graft loss among zero mismatched (3.5%) compared to six antigen-mismatched transplants (11.3%) and that there was an incremental increase in risk of graft loss with each increase in the number of mismatched antigens. Also, they showed that mismatches of one or two antigens with azathioprine treatment had better graft survival than six antigen mismatches treated with MMF. As noted above, Opelz and Döhler (4) examined the impact of HLA in two different decades (1985–1994 and 1995–2004) and saw that the impact was diminished in the second decade, but was still strong. Martins et al. (57) also saw a diminished impact of HLA matching with triple therapy of MMF, antithymocyte globulin, and tacrolimus. However, from the more current references cited above, it is apparent that HLA matching still provides the benefits of longer graft survival and better graft function.

### Opportunity for Transplantation and Allocation of Deceased Donor Organs

Although transplanting well-matched organs is highly desirable, the high degree of polymorphism in the HLA system results in a low likelihood of finding a well-matched, unrelated donor even when only HLA-A, -B, and -DR are considered (58). This is particularly true where there is great HLA heterogeneity, as in the United States. This is born out by the distribution of zero-mismatched deceased donor renal transplants in the United States during the period 1998–2001. The percentage of each group receiving a zero A, B, and DR mismatch was 21.4% of Whites, 7% of Blacks, 14.3% of Hispanics, and 6.6% of Asians (59). Considering only partial matching such as for HLA-DR, Vu et al. (60) determined that the average probability of finding a zero DR mismatch among local donors was 5% and this value was reduced to 2% when ABO compatibility was considered. Of course, the probability of a well-matched donor among first degree relatives is appreciably higher; however, this opportunity is not available to the vast majority of patients who need a kidney transplant. The desire for a well-matched donor should be balanced with a reasonable probability of finding such a donor. Algorithms for calculating the frequency of a donor who is a zero mismatch at a single locus or at any two or more loci are shown below (61). It is necessary to have allele and haplotype frequencies to perform the calculations, which can be found at various web sites. For frequencies for deceased donors where the donor population is comprised of different ethnic groups, the calculations should be done for each group that is a substantial proportion of the population. The values for each group should be weighted according to their proportion in the donor population and the weighted values should be summed to derive the probability of a donor in the total population.

#### Frequency of a Donor Who Is a Zero Mismatch at a Single Locus

This is a relatively easy calculation but must use allele, not antigen, frequencies. If the patient is homozygous for an antigen then, if *i* represents the frequency of the patient’s allele for the homozygous antigen, the frequency of a donor who is a zero mismatch at that locus is *i*^2^.

If the patient is heterozygous for an antigen and the frequencies of the patient’s alleles at that locus are given by *i* and *j*, then the frequency of a zero mismatch at one locus is

$$\begin{array}{cc} P\left(\text{zero single locus mm}\right) & =i^{2}+2ij+j^{2}\\ & ={\left(i+j\right)}^{2}. \end{array}$$

Note that there may be several alleles that encode a serologically defined antigen. Then the allele frequency for that antigen would be the sum of all the alleles that encode that antigen and to which the patient does not have antibody.

#### Frequency of Donor Who Is a Zero Mismatch at Two or More Loci

If the patient is homozygous at all loci under consideration, the probability of a zero mismatch is *h*^2^ where *h* is the frequency of the haplotype comprised of the loci under consideration.

If the patient is heterozygous at one or more loci then

1. Determine all the haplotypes that can be included in the phenotype of the loci under consideration.
2. Assign a population frequency to each haplotype, represented by *hn*.
3. The phenotype that has no mismatched antigens occurs when any one haplotype is in the homozygous state or with combinations of any of two of the compatible haplotypes.
4. This is given by *h*1^2^ + *h*2^2^ + *h*3^2^ + …2*h*1*h*2 + 2*h*1*h*3 + … which reduces to (*h*1 + *h*2 + *h*3…)^2^

Example: frequency of a zero B, DR mismatch for a patient with the phenotype A1, A2; B8, B44; DR11, DR17. The haplotypes that have no mismatched antigens are B8/DR11, B8/DR17, B44/DR11, and B44/DR17 that will have frequencies represented by *h*1, *h*2, *h*3, and *h*4, respectively. Then,

$$\begin{array}{cc} P\left(\text{zero B}/\text{DR mismatch}\right) & =h1^{2}+h2^{2}+h3^{2}+h4^{2}+2h1h2+\;2h1h3+2h1h4+2h2h3\\ & \;+\;2h2h4+2h3h4\\ & ={\left(h1+h2+h3+h4\right)}^{2}. \end{array}$$

The probability can then be used to determine the number of donors needed to achieve a certain probability of finding such a donor. This is determined by

$$P(\text{donor with selected phenotype among}\;n\;\text{donors})=1-{\left(1-y\right)}^{n},$$

where *y* is the probability a donor will have the selected phenotype and *n* is the number of donors,

$$\begin{array}{ll} {\left(1-y\right)}^{n} & =1-P(\text{donor with selected phenotype among}\;n\;\text{donors})\\ n\;\text{log}\left(1-y\right) & =\text{log}\left[1-P\left(\text{donor with selected phenotype among}\;n\;\text{donors}\right)\right]\\ n & =\text{log}\left[1-P\left(\text{donor with selected phenotype among}\;n\;\text{donors}\right)\right]/\log\left(1-y\right). \end{array}$$

For example, when the frequency of donors with the selected phenotype is 0.01, the number of donors, *n*, needed to achieve a 95% probability of such a donor occurring is

n=log 0.05/log0.99=298.

When trying only to avoid unacceptable mismatches, the frequency of donors can be obtained from programs such as the UNOS CPRA calculator, which can be found at https://optn.transplant.hrsa.gov/resources/allocation-calculators/cpra-calculator/. The CPRA calculator determines the frequency of donors with unacceptable antigens using allele and haplotype frequencies in the United States donor population. The probability of a donor with no unacceptable antigens among *n* donors is given by

$$P\left(\text{donor with no unacceptable antigens among}\;n\;\text{donors}\right)=1-{\text{CPRA}}^{n}.$$

Following the derivation above,

n=log[1−P(donor with no unacceptable antigens)]/log CPRA.

So, for a patient with a CPRA of 0.95, the number of donors needed to have a 95% probability of finding such a donor is

n=log0.05/log0.95=58.

## HLA Mismatches and Sensitization

Sensitization to HLA antigens can be provoked by transfusion, pregnancy, or transplantation. Of these, the rate of sensitization and the strength and duration of HLA antibodies is greatest for transplantation where more than 70% of transplantation patients become sensitized compared to approximately 40% of transfused patients and 11–19% of parous females (62). We previously examined the impact of varying degrees of mismatch for HLA-A, -B, -DRB1, -DRB3-5, and DQ, for a possible total of 10 mismatches, among 534 renal transplant patients (63). We found that the rate and extent of sensitization was proportional to the degree of mismatch. There was a substantial increase in extent of sensitization, on average, for patients whose previous transplant involved mismatches of two or more antigens, regardless of the race, gender, or previous sensitization status of the patient. Wait list time is longer for sensitized patients than for non-sensitized patients and this incurs greater costs for dialysis and antibody testing. For patients on the waitlist in 1996 and 1997, we determined that the costs were $297,204, $480,803, and $1,036,078 for patients with PRAs of 0–9, 10–79, and ≥80. These figures are likely to be much higher today with higher dialysis costs and more sensitive antibody tests, even when antibody testing frequency is reduced as a cost-saving measure. Thus, the more mismatches, the greater the risk of sensitization and the higher the cost of a subsequent transplant.

Willicombe et al. (64) looked at the *de novo* development of donor-specific antibody among 505 renal transplant recipients who had no pretransplant donor specific antibody when tested in multianalyte bead assays on the Luminex^®^ platform. They found that 18.2% of patients made donor-specific antibody after transplantation. Of those, 30% were specific only for class I, 45% only for class II, and 25% for both. Interestingly, half were specific only for DQ. The frequency of *de novo* donor-specific antibody among patients matched for 2DR vs. 2DQ antigens was 9.4 and 21%, respectively. In a smaller study, Tagliamacco et al. (65) found an even higher rate of posttransplant antibody following a first transplant in 82 non-sensitized pediatric renal transplant patients. In this study, 29% made donor-specific antibody *de novo*, 83% of which was specific for DQ. Similar to the findings of Lopes, Kosmoliaptsis et al. (66) saw that 67% of previously transplanted patients were sensitized. When examined by class of antigen mismatched, they found that HLA-A mismatches had a greater effect than either HLA-B or -C. For class II antigens, the effect was comparable for DR and DQ but greater than for class I with the resultant antibodies stronger than those for class I. Among patients who were sensitized prior to the initial transplant, the frequency of sensitization went from 13 to 34% for class I mismatches and from 5 to 22% for class II mismatches. Meier-Kriesch et al. (67) looked at sensitization among nearly 16,000 patients who were relisted after the loss of a first graft. They found that increases in PRA and the odds of being newly sensitized were proportional to the number of previously mismatched HLA-A, -B, and -DR antigens. They saw a strong effect of mismatches of HLA-A and -B with two HLA-A mismatches producing a greater increase in PRA than did two HLA-B mismatches (23 vs. 13%). Among patients with no HLA-A, -B mismatches, only 10% were newly sensitized upon being relisted but that increased to 50% for mismatched patients. Furthermore, they saw a greater increase in sensitization in Blacks compared to Whites (18.3 vs. 13.9%). This has implications for changes in United States deceased donor allocation policies as eliminating points for HLA-B matches has resulted in an increase in the percentage (48.1–52%) of deceased donor transplants going to Blacks with a concurrent increase in the frequency of two HLA-B antigens mismatched (46–72%) (68). In turn, this increased level of mismatch may drastically decrease the opportunity for a subsequent transplant. Evidence for this was presented in a report by Gralla et al. (69) who looked at data for nearly 12,000 pediatric renal transplant patients, 2,704 of whom experienced graft failure and were listed for another transplant. There were 1,847 who were retransplanted. Among patients who were retransplanted, the mean PRA had increased from 6 to 45% while among the 857 who did not receive another transplant, the mean PRA increased from 8 to 76%. The ability to obtain a subsequent transplant was inversely correlated with the number of previous mismatches. Eighty percent of patients whose first graft was mismatched for two or fewer antigens were retransplanted. The percentage dropped to 56% for more than three previous mismatches. In a similar study of 8,433 pediatric patients, Foster et al. (70) also saw a declension in the likelihood of a second transplant with increased numbers of mismatched antigens in a first graft.

The conundrum created by these data is that on the one hand, higher numbers of mismatches not only reduce graft life and function but also increase the risk of sensitization with a resultant decreased opportunity for a future transplant, which may have a greater impact on pediatric patients who will most likely need more than one transplant. On the other hand, the opportunity for finding a well-matched unrelated donor is small for the majority of patients and extended time waiting incurs increased morbidity and mortality. A compromise may be to select donors with mismatches that have a low probability of inducing a humoral response. It has been proposed that antibody response to a transplant correlates with epitope load presented by the donor HLA antigens or the number of amino acid differences in the membrane distal portions of donor and recipient HLA antigens and great interest has been generated in “epitope matching” (71–74). Epitope matching may be the answer to reduce the humoral response to mismatched grafts, however, greater elucidation of the antibody binding and immunogenic properties of proposed epitopes is needed before widespread clinical application is possible. The portion of the HLA molecule seen by the patient’s immune system is comprised of its two membrane distal domains. Epitopes may reside on the α helix of one or the other domain or may be formed by interaction of the two α helices. This was demonstrated clearly in exon shuffling experiments (75–77). The properties, such as electrostatic potential and hydrophobicity, of the amino acids that comprise an epitope affect the identity and immunogenicity of the epitope and must also be taken into consideration (78, 79). Improvements in HLA antibody identification have permitted serologic confirmation of several proposed epitopes. However, the sera of most patients contains a mixture of antibodies and it is often difficult to define epitope specificity precisely.

We investigated antibody specificity in 703 patients who developed antibody to donor HLA following the transplantation (80). This was not a study of the frequency of antibody development. The hypothesis was that if all HLA antigens are equally immunogenic, newly developed antibody should be specific for all the mismatched donor antigens. We found that the frequency of antibody response varied both among loci and among the different antigens at each locus (Table 2). We also found that the presence of an antigen in the patient’s phenotype that was cross-reactive with the mismatched donor antigen reduced the response to that antigen. The strength of the effect of a cross-reactive antigen in the patient’s phenotype varied among antigen pairs. An HLA-A1 in a patient diminished the response to an HLA-A3 mismatch by 44%, but an HLA-A11 in the patient diminished the response to HLA-A3 by only 19%. Interestingly, not only was there was a variability in the effect of cross-reactivity but also there was a directionality. For example, an HLA-A2 in the patient reduced the response to HLA-B57 by 83%, but an HLA-B57 in the patient reduced the response to HLA-A2 by only 8%. Antibody response was not affected by the total number of mismatches, the number of mismatched DR antigens, nor the DR phenotype of the patient. These data could be used to identify the donor who would be the least likely to provoke a humoral response when several donors are available. Two groups of patients in particular may derive a greater benefit from the consideration of the immunogenicity of donor antigens. Pediatric patients are likely to need more than one transplant in their lifetime and when well-matched donors are not available, it would be possible to use these data to limit the sensitization to the first transplant. Black patients have higher rates of sensitization than do White patients. As of 2013, the percentage of different groups of patients on the United States wait list who are sensitized was 43, 35, and 33% for Blacks, Whites, and Hispanics, respectively (81). We also found a higher antibody response in Black vs. Whites in our study of immunogenicity, although the difference was not statistically significant. The racial disparity in deceased donor transplants in the United States has been attributed to the use of HLA matching in allocation schemes. However, this disparity is also due in part to a higher rate of sensitization among Black patients and to differences in the ABO distribution between the donor population and Black patients (82). Unfortunately, the utility of data on immunogenicity would be limited with deceased donor allocation schemes that are driven primarily by wait time.

**Table 2:** **Frequency of response to mismatched HLA antigens**.

Response^a^	HLA-mismatched antigen			
	A	B	DR	DQB
Mean overall	53.2	42.4	52.6	59.0
Range^b^	30.8–76.2	15.0–66.1	40.0–73.0	47.4–90.0
Mean with no cross-reactive antigen in patient	60.2	52.0	61.0	71.3
Mean with cross-reactive antigen in patient	49.7	35.5	43.0	45.5

*Frequencies of antibodies, defined by multiplexed bead assays, to mismatched HLA antigens were determined from 703 renal transplant patients who had no detectable donor-specific antibody before transplantation. The impact of patients having antigens in their phenotype that were cross-reactive with the mismatched antigen was also assessed. Adapted from Lucas et al. (80)*.

*^a^Percentage of mismatched patients who made antibody*.

*^b^The range of antibody response to different antigens within the locus*.

## HLA-Matching Strategies for Sensitized Patients

Although it may be desirable to avoid all donor-specific antibodies, this will prevent many patients from being transplanted. Several strategies have been developed to deal with sensitization. It is important to correctly identify all the HLA antibodies of a patient, assess the level of risk associated with those antibodies, determine the level of risk that is acceptable, and determine the likelihood of finding a suitable donor, that is a donor who meets the risk specifications.

Solid phase immunoassays (SPI) and, in particular, the multianalyte bead assay performed on the Luminex^®^ platform provide outstanding sensitivity and specificity in HLA antibody detection and characterization. These assays have provided improved detection of antibodies specific for HLA-C, -DQ, -DP, and subtypes of serologic antigens defined by alleles within an antigen group. They are essential to the identification of epitopes and reveal low-level sensitization not detected in cell-based assays. Utilization of SPIs is essential for safe transplantation of the sensitized patient, but accurate interpretation of results requires substantial experience and expertise. The high degree of sensitivity of these assays make them subject to interference from IgM, complement, and immune complexes (83), and the presence of distorted HLA molecules in the assay may lead to incorrect positive or negative results (84). SPIs are semiquantitative and should be used in conjunction with cell based assays (85) and the results correlated with crossmatch test results (86, 87). Tambur et al. (88) have shown that titrating sera in the multianalyte bead assay provides a good indication of antibody strength and is very useful when cell-based assays cannot be performed either because of lack of donor cells or because of the presence of therapeutic cell depleting agents in the serum. One of the most difficult problems is determining a threshold for positivity – that is, knowing when an antibody is really present. Although manufacturers have greatly reduced lot-to-lot variability in sensitivity, the high sensitivity of these assays makes them susceptible to run-to-run and operator variability. Furthermore, there is bead-to-bead variability due to varying amounts of misformed molecules on different beads and greater antigen concentration on beads bearing HLA-C, -DQ, and -DP antigens. We have found that these problems are diminished somewhat with phenotype panels, but these panels are not sufficiently informative for broadly reacting sera. Using cutoffs for positivity that are too low may deprive some patients of safe transplants while cutoffs that are too high can represent an unrecognized risk.

There is a great deal of information about antibodies to HLA-A, -B, and -DR and data about antibodies to other HLA antigens are increasingly available. SPIs have shown that antibodies to DQ are inordinately common following transplantation and their complexity is being increasingly appreciated (25, 26). As noted earlier, because both polypeptide chains of DQ molecules are polymorphic analysis of DQ reactive antibody must take into account both the DQA and DQB alleles. Antibodies to a unique combination of DQA and DQB are most readily recognized when a patient’s antibody reacts with a molecule bearing the patient’s own DQB but a different DQA and does not react with other DQ molecules bearing the same DQA or when the antibody reacts with only one molecule bearing a particular DQA and DQB but with no other molecules bearing either that DQA or DQB. Less is known about antibodies to HLA-C and DP. As early as 1986, hyperacute rejection of a renal allograft due to antibody to an HLA-C antigen was reported (89). More recently, Bachelet et al. (90) reported on loss of a renal graft they attributed to antibodies to two donor Cw antigens. Although the flow cytometric crossmatch was positive, the mean fluorescence intensity (MFI) values were moderately low (6,931 and 8,920). The patient also had antibodies to donor DP antigens. Ling et al. (91) reported on eight patients with antibody to donor Cw antigens, one of which had a positive FCXM while the crossmatch tests of the other seven were negative. The patients were followed for 3–24 months during which there was no antibody-mediated rejection and no graft loss. While exceptional cases of acute rejection mediated by antibodies to HLA-C may occur, the inherently low expression of these antigens suggests that they may be more involved in chronic rejection (92).

There is complexity with DP antibodies that is the result of cross-reactivity between certain HLA-DR and certain HLA-DP antigens due to shared epitopes (93). Two sequence dimorphisms of DPB1 define the immunodominant serologic epitopes of HLA-DP. Callender et al. (94) showed that while 42% of 650 patients on a renal waiting list had DP antibody, nearly 80% had antibody to the cross-reactive DR antigens. The strengths of most of the antibodies was low with only 3 of the 271 sera yielding a positive cytotoxicity crossmatch. Furthermore, 40% of patients with DP antibody had not been previously transplanted. These data suggest that much of DP reactivity may be cross-reactivity with DR which may account, to some extent, for the reduced graft function and survival associated with DR mismatching. What needs to be determined is the extent to which DP antibody alone is pathogenic. Jolly et al. (95) reported two cases of antibody-mediated rejection and graft failure due to antibody to donor DP. In neither case was there antibody to other donor antigens, nor did the donors have DR antigens cross-reactive with the DP antibodies, suggesting that the graft failure was attributable to the DP antibodies. Redondo-Pachon et al. (96) observed higher rates of acute rejection and of delayed graft function when donor-specific antibodies included specificity for DP. Goral et al. (97) also reported antibody-mediated rejection in two patients who received kidneys from donors mismatched only for DP and who had flow cytometric positive crossmatches positive with donor B cells and negative with autologous B cells. Collectively, the data indicate that patients should be tested for antibodies to all expressed HLA loci – A, -B, -C, -DRB1, -DRB3-5, -DQA, -DQB, -DPB and most likely -DPA and that both donors and recipients should be typed for these loci.

Economic pressures have forced many transplant programs to reduce the amount of antibody testing performed especially for patients who are likely to wait a long time to transplantation. HLA antibodies can be transient, particularly those produced in response to transfusion or pregnancy. In the absence of a complete screening history, it is possible that sensitization would not be recognized. The amount of risk associated with donor-specific antibody that is present historically but not currently has not been clearly resolved. Some reports indicate that renal transplantation could be performed safely in the face of an historic positive, current negative crossmatch (98, 99). Lyne et al. (100) reported on 47 patients with positive historic, current negative crossmatches. However, only 18 of the 47 crossmatches remained positive after treatment of the serum with dithiothreitol, indicating that 29 of the positive crossmatch results were due to IgM antibody. Overall graft survival rates for patients with apparent IgG antibody were not significantly different from those with apparent IgM antibody. In contrast, Leavey et al. (101) saw increased early acute rejection among patients with historic IgM (42%) or IgG T cell positive crossmatches (57%) compared to patients with historic B cell only positive crossmatches (32%) and the IgG-positive group also had reduced 1-year graft survival (71%) compared to the other two groups (95%). Using a method developed in our laboratory to enumerate HLA-specific B cells by staining B cells with HLA tetramers, we found that patients with an increased level of B cells specific for HLA-A2, -A24, or -B7 who did not have antibody to those specificities at the time of transplant made antibody specific for the HLA antigen for which they had increased level of B cells even if the transplant was not mismatched for those antigens. Patients without increased numbers of HLA-specific B cells did not make the antibody (102). One patient with elevated B cells specific for HLA-B7 was mismatched for the antigen. That patient made IgG antibody to B7 within 48 h of transplantation and experienced severe antibody-mediated rejection. The timing of the antibody appearance indicates an anamnestic response. Donor-specific antibody of the IgG class that appears within the first posttransplant week reflects an anamnestic response that indicates a risk for patients with cryptic sensitization. A possible explanation for the apparently conflicting results cited above is that in some cases, the disappearance of antibody reflects a senescence of the immune response, while in others, it indicates an active suppression. Another possibility is that certain immunosuppression agents abrogate an anamnestic response. We studied the effect of rituximab treatment on posttransplant antibody responses in 26 patients who had elevated HLA-specific B cells, but no antibody specific for the tested antigen at the time of transplant. Of patients treated with rituximab, 0 of 10 made antibody after transplantation while 13 of 16 who were not treated with rituximab did make antibody (103). These data suggest that a positive historic crossmatch or known previous sensitization represents a manageable risk that does not require avoiding those antigens to which a patient was previously sensitized.

Strategies for transplanting sensitized patients include avoiding mismatches to which the patient currently has antibody, overcoming low-level donor-specific antibody with more intense immunosuppression, or eliminating or reducing donor-specific antibody to an acceptable level *via* desensitization applied prior to or at the time of transplantation. It is likely that no one approach is optimal for all patients and that transplanting sensitized patients in a timely and safe manner may require programs to utilize all three strategies, customized to the immune status and medical condition of each patient.

Finding donors to which a sensitized patient does not have antibodies has been greatly enhanced by kidney paired donation programs. These programs are directed toward patients who have a willing, but incompatible living donor. By transporting donor kidneys, recipients and donors can undergo surgery at their home institution. Another approach, the acceptable mismatch program, has significantly increased transplantation rates for patients awaiting a deceased donor transplant. This program was pioneered by Claas and colleagues in the Netherlands in the late 1980s (104). As initially implemented, the strategy was the determination of HLA-A and -B mismatches to which the patient had not formed alloantibodies. Successful implementation in Eurotransplant involved extensive antibody screening for HLA-A and -B specific antibodies coupled with sharing of sera among participating centers for crossmatching of all ABO-compatible donors. Allocation within Eurotransplant for the acceptable mismatch program affords highly sensitized patients the highest priority when a donor becomes available who is compatible with the patient’s antibody profile (105). Since its implementation in Eurotransplant, waiting time among highly sensitized patients has been significantly reduced while both short- and long-term graft survival comparable to non-sensitized patients has been achieved (105, 106). Use of current SPIs for definition of HLA-specific antibodies, coupled with a molecularly based algorithm for determination of acceptable antigen mismatches has added to the potential application of acceptable mismatch programs (107, 108). In a cost-benefit analysis among patients on the deceased donor wait list in Australia, an acceptable mismatch allocation model was found to be an equitable approach to improve access for highly sensitized transplant candidates without compromising the benefits to other patients on the wait list (109). Acceptable mismatch programs have the advantages of being lower in cost and non-invasive compared to desensitization protocols; however, a compatible donor may not be found for up to 40% of patients who may have rare HLA phenotypes and/or be very broadly sensitized (105, 110). The degree of HLA heterogeneity among the patient population compared to the donor pool is a factor in the United States with large numbers of Black transplant candidates, as the degree of HLA phenotype heterogeneity is significantly higher among Blacks than among other ethnicities (58). Therefore, it has been recognized that for successful transplantation of highly sensitized patients, both acceptable mismatch programs and desensitization should be considered (110).

Low-level, donor-specific antibody is associated with an increased frequency of antibody-mediated rejection (111–113) and subclinical rejection (114) and reduced long-term graft survival (115, 116). However, despite efforts to avoid mismatches with a donor to whom a patient has antibodies, there remain patients for whom such a donor cannot be found. Dialysis reduces quality of life, is attended by numerous health issues particularly in young patients, and limits many activities enjoyed by healthy individuals. Ameliorating or delaying the effects of donor-specific antibody can be achieved with various therapeutic agents and procedures such as lymphocyte and plasma cell-depleting agents, plasmapheresis, and intravenous immunoglobulin. It has been clearly demonstrated both in a single center (117) and a multicenter study (118) that desensitization provides a significant survival benefit over patients receiving a compatible deceased donor transplant or patients who remain on a wait list. Eliminating unacceptable antigens or antigens to avoid can be done by raising the threshold for what is unacceptable, without consideration of specificity or the breadth of sensitization. In an Australian kidney paired donation program with a registry of 53 donor–recipient pairs and two altruistic donors, no matches were found using a cutoff of 2,000 MFI for acceptability. When the threshold was raised to 8,000, matches were found for 70% of the patients (119). The threshold for unacceptable antigens could be changed according to the correlation with crossmatch. That is, the threshold could be raised to just below what would yield a positive flow cytometric crossmatch. This may be more difficult to assess for donors to whom a patient has multiple antibodies as the collective strength of the antibodies is difficult to assess from SPIs. One may choose to eliminate unacceptable antigens by specificity or by source of sensitization. For example, one may choose to keep as unacceptable, antigens that were previous transplant mismatches and to which the patient has antibody at a low level. Ferrari et al. (87) have recommended raising unacceptable thresholds only if desensitization treatment is available and if the antigens are not rare in the donor population. Using allele and haplotype frequencies or programs such as the CPRA calculator, one can determine the impact on the likelihood of finding a donor when unacceptable antigens are eliminated (shown above). Table 3 provides the number of unrelated donors needed for a 95 or 99% probability of finding a donor for different levels of CPRA.

**Table 3:** **Number of donors needed for different CPRA levels**.

CPRA^a^	Number of donors required	
	95% probability of a donor^b^	99% probability of a donor^b^
0.9999	29,956	46,049
0.9990	2,994	4,602
0.9900	298	458
0.9500	58	90
0.9000	22	44
0.8500	18	28
0.8000	13	21
0.7500	10	16

*The numbers (*n*) of donors required for 95 and 99% probability (*P*) of finding a compatible donor at different CPRA levels are shown. The numbers were derived by the following algorithm: *n* = log[1 − *P*(donor with no unacceptable antigens)]/log CPRA*.

*^a^This is also 1- frequency of donors with no unacceptable antigens (see text)*.

*^b^Number of donors, rounded to nearest whole number, needed to have a 95 or 99% probability of an acceptable donor*.

One may eliminate unacceptable antigens by either class I or class II based on the differential expression of these antigens. Muczynski et al. (120) reported that class II antigens were expressed constitutively in the endothelium of renal peritubular and glomerular capillaries. However, McDouall et al. (121) demonstrated that class II was not expressed constitutively on large vessels. Several others have reported that cultured endothelial cells do not express class II constitutively (122–124). It is difficult to know if cultured cells are representative of the *in vivo* situation or if cells obtained *via* biopsy have been provoked to express class II. Our experience with desensitization indicated that patients with persistent DR or DQ antibody at a level below flow cytometric crossmatch had only a slightly increased frequency of antibody-mediated rejection compared to patients with no detectable donor-specific antibody and there was no increased rejection in patients with persistent antibody to DR51, 52, or 53 (125). In fact, 10-year graft survival occurred with one patient who had persistent antibody to donor DR52 at the level of cytotoxicity. Antibody-mediated rejection occurred only when the patient was treated with thyroxine, an agent known to stimulate class II expression. Another patient had graft survival of at least 5 years with a DQ7 antibody that had spiked to a very high titer in the cytotoxicity assay following an anaphylactic reaction. These data and examples suggest that if HLA class II antigens are expressed constitutively on vascular endothelium, it is at low levels.

Thus, patients with levels of antibody that are naturally low or have been reduced by desensitization are able to be transplanted with reasonably good graft function and survival. However, these patients should be monitored frequently in the early posttransplant period and periodically for the life of the graft for changes in antibody level. Pro-inflammatory events, such as infection, trauma (such as surgery), an allergic reaction, and blood transfusion, can all stimulate non-specific activation of memory B cells leading to an increase in donor-specific antibody (126, 127). Finally, although not the topic of this review, it is worthwhile to mention that a very reasonable approach to transplanting the sensitized patient is with a donor who is well matched for HLA, but is ABO incompatible. This may be particularly beneficial to the pediatric transplant candidate (128).

## Summary

We have reviewed data here that are summarized as follows:

- All HLA mismatches are associated with some degree of risk of reduced graft function and survival and the risk is proportional to the number of mismatched antigens.
- Good HLA matches with unrelated donors are uncommon, and the desire to achieve a good match should be balanced against the risk associated with prolonged time on dialysis.
- Repeated mismatches represent an increased risk only in sensitized patients or in patients who underwent nephrectomy of a previous graft.
- There are other deleterious effects of mismatching, one of the most serious being sensitization, which is most problematic for patients who will need another transplant.
- Balancing risk of sensitization and wait time may be achieved by favoring less immunogenic mismatches.
- Matching strategies for sensitized patients may be to avoid donor antigens to which a patient has antibody or to reduce antibody strength to an acceptable level and/or utilize more intense immunosuppression.
- Matching strategies should be customized to both the patient and to the transplant program’s resources.

## Author Contributions

AZ and ML together developed the outline for this paper, reviewed all relevant literature, wrote the paper, and prepared the figure and all tables.

## Conflict of Interest Statement

The authors declare that the research was conducted in the absence of any commercial or financial relationships that could be construed as a potential conflict of interest.

## Abbreviations

CPRA, calculated panel reactive antibody; FCXM, flow cytometric crossmatch; PRA, panel reactive antibody; SPI, solid phase immunoassay; USRDS, United States Renal Data System.
